# Saliva between normal and pathological. Important factors in determining systemic and oral health

**Published:** 2009

**Authors:** Gabriela Iorgulescu

**Affiliations:** * "Carol Davila" University of Medicine and Pharmacy, Bucharest

## Abstract

There is a tendency in current medical research to explore the importance and symptomatology of saliva. The question to which increasingly more researchers from the medico-legal, systemic and dental fields tried to answer and bring together arguments for a greater emphasis is referring to the role of saliva in the health of the patient.

Up until our time, people have looked at the importance of saliva from another perspective: saliva helped in pasting envelopes or stamps, or mostly in reported cases of public speakers faced with the impossibility of having a coherent speech due to sensations of dry mouth.

This ‘dry mouth’ condition, named xerostomia in medical terms, has been used since antiquity as a test in detecting lies, knowing since then that the inhibition of emotional salivary glands, the feeling of ‘dry mouth’ is caused by anxiety, thus being a potential incrimination.

Although hundreds of publications have insisted on the etiology and complications of the salivary gland hypofunction, only a few health professionals used to harvest saliva tests.

As in the case of urine and blood, saliva quality and quantity are affected by a multitude of medical conditions and treatments, as well as the patient's psychological state.

A review of the formation, function and dysfunction of salivary glands may convey the significant role played by saliva in health and disease, especially in detection and recognition of salivary gland hypofunction, systemic disease, and the psychological states, and thus prevent complications caused by these conditions.

## Study of the Function and Dysfunction of Saliva

Saliva Formation

Saliva is produced by three pairs of major glands and numerous minor salivary glands located in the oral cavity. The parotid, submandibular, and sublingual salivary glands contribute to 90% of total saliva secretions, while minor salivary glands contribute to the remaining 10%. The amount of saliva secreted by the major and minor glands is referred to as whole saliva. In the resting (unstimulated) state, approximately two-thirds of the total volume of the whole saliva is produced by submandibular glands. Upon stimulation, the parotid glands are responsible for at least 50% of the total volume of saliva from the mouth. Sublingual glands contribute to a small percentage, both in the unstimulated or stimulated states of the salivary glands. Minor salivary glands contribute significantly to the lubrication of the oral mucosa because of their high protein content. Unlike some other minor salivary glands which are composed exclusively of mucous cells, parotid glands are serous and produce water like secretions. Submandibular and sublingual glands are mixed.

In general, acinar (secretory) cells are responsible for the production of the primary saliva. The ductal cells are responsible for further modifications of saliva until it is secreted in the mouth. Saliva is 99% water and 1% protein and salts. The normal daily production of saliva varies between 0.5 and 1.5 liters. The whole unstimulated saliva flow rate is approximately 0.3-0.4 ml / min. This rate decreases to 0.1 ml / min during sleep and increases to about 4, 0-5, 0 ml / min during eating, chewing and other stimulating activities. Saliva is always hypotonic to plasma. As the whole saliva flow rate will increase, the tonicity of the saliva will increase too. Salivary glands secretion is mainly controlled by the autonomous nervous system. Parasympathetic stimulation produces abundant quantities of watery saliva, whereas sympathetic stimulation produces more viscous saliva (Bardow, Nauntofte and Pedersen, 2004).

***Saliva Function***

Saliva plays a significant role in the protection of the intraoral structures against injuries caused by various pathogenic microbes, mechanical or chemical irritants. 

The functions of the saliva:

- Defensive/buffering capacity 

- Remineralization of teeth

- Restoration of soft tissues 

- Lubrication capacity 

- Digestion 

- Antimicrobial capacity

Saliva contains three buffer systems (bicarbonate, phosphate and protein) and helps in maintaining acceptable pH range of 6.0-7.5 within the mouth. When a substance is placed in the oral cavity, the flow of saliva will increase depending on its taste, consistency and concentration. When the volume of saliva is approximately 1.1 ml, a swallowing reflex is triggered. Salivary stimulation, dilution of tasting and swallowing will continue until the concentration of the tastings reaches a point where it ceases to stimulate salivary flow. The oral clearance of various substances will be prolonged in the absence of saliva, resulting in possible harm to intraoral hard and soft tissues. Under normal physiologic conditions the saliva is oversaturated with calcium hydroxyapatite, which prevents dental demineralization. In addition, the salivary protein pellicle protects the teeth against irritants.

Human saliva contains α amylase and lipase, substances that may play a role in starch digestion and decomposition triglyceride breakdown in neonates with pancreatic dysfunction. Salivary mucins play a significant role in lubricating the intraoral structures and help forming a barrier against microbial invasion. Lysozyme and lactoferrin are examples of proteins with antimicrobial properties. Lactoferrin is believed to have antibacterial, antifungal, and antiviral properties. Salivary peroxidase has antibacterial properties, whereas histatins have been associated with antibacterial and antifungal properties. Salivary epidermal growth factor enhances the speed of the oral mucosal healing and protects the esophageal mucosa. In addition to these proteins with specific functions, other enzymes could serve as indicators in diagnosis, such as pseudo cholinesterase for mental disorders (Giddon and Lisanti, 1962). Saliva contains other organic components, such as glucose, urea, cortisol, sex hormones and blood group substances, which have also been utilized in saliva as screening/diagnostic tools.

***Saliva Dysfunctions***

Saliva quantity and quality can be affected by multiple diseases and medical treatments. Salivary cortisol level is increased as a response of the adrenal cortex to stressors such as chronic dental anxiety, stressful activities in front of computer, viewing anxiety-inducing videos and masticator muscle activity caused by clenching teeth. Relaxation methods such as viewing soothing videos, listening to music (*Music Therapy*, Iamandescu, IB, 1997), may lower the saliva cortisol and amylase levels. As noted earlier, the feeling of dry mouth may have a psychological cause. Psychological processes are often accompanied by disturbed oral sensations, and, in fact, most individuals have experienced a sensation of dry mouth during a period of acute stress. Along with depression, mental stress is sometimes associated with a dry mouth condition, either as a result of the disease itself or as an adverse effect of drugs used in management of the psychological state (Bergdahl and others, 1997; Bolwig and Rafaelsen, 1972; Daviessi Gurland, 1961).

These issues were highlighted in the *Burning Mouth Syndrome* - BMS; a condition regarded, along with bruxism, as psychosomatic condition of the oral area, its symptoms matching the differential diagnosis of salivary gland dysfunction.

The burning mouth syndrome is a set of painful and burning sensations in the mouth experienced even when clinical investigation of the mucosa proves to be normal. BMS incidence is 3% of the population (Mott, Grushka & Sessa 1993) and patients are often surprised that others experience that condition too, because they do not have a clear knowledge of this disease. It is assumed that a large number of agents can be responsible for this condition: 

- Local (e.g., dental materials used to restore teeth)

- Systemic (including lack of minerals, vitamins, etc.).

- Stressful life events.

- Mental health problems.

- Psycho-social difficulties 

Studies of cortisol levels in depressed patients have led to interesting results provided that the technical aspects of the steroid sampling are controlled. There seems to be differences in salivary cortisol between patients with endogenous and nonendogenous depression (Iorgulescu, 2006) generally, there is a correlation between plasma ACTH levels and salivary cortisol, but this relationship is not present in patients with endogenous depression, suggesting either an effect of medication or a disorder of regulation of cortisol secretion (Galard and others, 1991). Self-induced vomiting and binge eating are features of the bulimia nervosa. Saliva function has been studied in this group and it is known that approximately 25% were affected by sialadenosis (Riad, Barton and Wilson, 1991, Roberts and others, 1989). Some studies have shown that parotid function is reduced in bulimics, meaning that resting and stimulated salivary flow rates are reduced in patients with sialadenosis, and the total protein and amylase levels are increased. Other studies of the parotid and submandibular gland function have shown no differences in function in relation to controls, and amylase levels were equivalent.

Xerostomia is a common oral disease associated with more than five hundred medications (Sreebny and Schwartz, 1988). Polypharmacy is the most common cause of xerostomia (complaint of dry mouth) and salivary glands hypofunction (objective evidence of reduced saliva flow rate) in the elderly. The most common types of medication with xerogenic potential are those with anticholinergic and sympathomimetic actions. Salivary gland hypofunction is a condition most often overlooked; many patients who take xerogenic medications may not know that they are at risk of oral complications such as dental caries and fungal infections. Therefore, the absence of subjective complaints of dry mouth sensations does not indicate an adequate level of production of saliva. Accordingly, the diagnosis of drug- induced hyposalivation requires measurements of saliva output or flow rate. 

Besides oral medication with inhibitory effect on the quantity of saliva, other chemotherapeutic modalities such as chemotherapy or radiotherapy can result in quality and quantity changes. There is a correlation between the severity of salivary gland hypofunction and the degree of exposure to radiation. Xerostomia is one of the most common complaints for patients who have undergone radiotherapy and/or chemotherapy. 

**Assessing the patient with salivary dysfunction**

***Chronic Conditions Associated with Salivary Gland Hypofunction in Adults***:

Medication

- Antidepressants

- Antipsychotics

- Antihistamines

- Antiemetics

- Antiretroviral therapy (protease inhibitors)

- Decongestants

- Appetite suppressants

 Diuretics

Irradiation

Chemotherapy

Medical conditions

- Sjögren’s syndrome

- Viral infections (HIV, HCV)

- Uncontrolled diabetes

- Alzheimer’s disease

- Hypertension

- Depression

***Signs and Symptoms Associated with Common Chronic Salivary Gland Hypofunction:***

SIGNS

-Dry, chapped lips; desiccated, dry and fissured tongue

-Angular cheilitis / pseudomembranous and erythematous candidiasis

- Dental caries (cervical and root caries in particular)

- Gingivitis

SYMPTOMS

- None (often may be asymptomatic)

- Difficulties in swallowing, chewing, speaking

- Bad taste, breath

- Sore mouth, lips, tongue

- Burning sensations in the mouth, lips, tongue 

- Difficulty wearing removable intra-oral prostheses

- Frequent need to sip water for food

- Frequent awakenings at night with dry mouth

- Dry mouth, nose, and throat

**Fig. 1:**
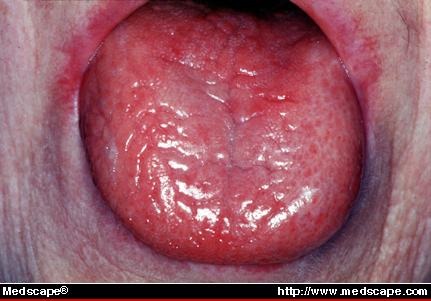


**Fig. 2:**
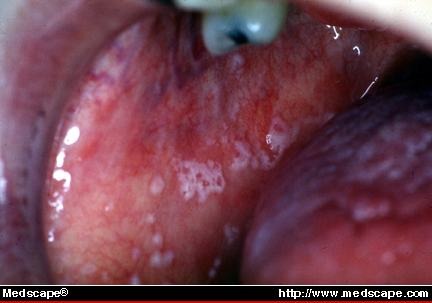


Acute pseudomembranous candidiasis. This SS patient has recurrent episodes of acute pseudomembranous candidiasis due to her extreme salivary gland hypofunction. (**Fig. 2**) 

Salivary levels of microorganisms (*Streptococcus mutants* and *Lactobacillus acidophilus*) and *Candida albicans* are usually used for assessing susceptibility to dental caries, and oral candidiasis, respectively (**Fig. 2**).

Evaluation of salivary gland function plays an important role in maintaining oral health and should be included in the first visit of each new patient, as well in the observations made during the subsequent visits. Regardless of subsequent complaints there are standard questions that can identify patients with a high risk of salivary gland hypofunction. The four most common questions are: 

1. Is the saliva flow rate too reduced, exaggerated or you can’t discern a difference?

2. Do you have difficulty swallowing? 

3. Do you experience a dry mouth sensation during your meals?

4. Do you sip liquids to help with the swallowing of solid food? 

**Figure:**
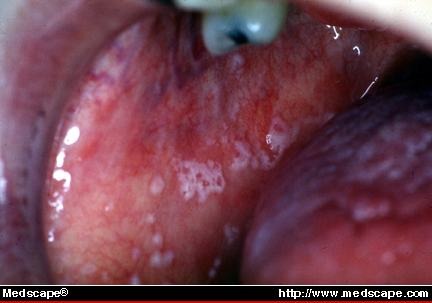
*Salivary gland enlargement. Facial asymmetry produced by enlargement of the right gland climax in a patient with SS. The swelling is asymptomatic and fluctuates in size throughout the course of several months (www.medscape.com)*.

## Current Diagnostic Values and Future Directions

Qualitative and quantitative changes of saliva may be used to detect exposure to chemicals and pathogens, or in quantifying the severity or susceptibility to different conditions (Kaufman & Lamster, 2002; Tabak, 2001). For example, the salivary levels of alcohol (ethanol), tobacco (nicotine, cotinine), cocaine, marijuana, opiate and methadone correspond well with their concentrations in serum and are used as screening tools by insurance companies and state institutions to assess exposure to these chemicals. The presence of antigens or antibodies of the pathogens in saliva, such as HIV-1, HIV-2, hepatitis A, B, C, measles, mumps, or rubella, may be used to evaluate possible exposure to these pathogens. Salivary levels of microorganisms (*Streptococcus mutants* and *Lactobacillus acidophilus*) and *Candida albicans* are usually used for assessing susceptibility to dental caries and oral candidiasis, respectively (Fox, 2004; Kaufman & Lamster, 2000). Saliva can also be used to monitor disease progression and the response to pharmacotherapeutic agents such as insulin, cortisol, aldosterone, estrogens, progesterone, lithium, theophilline, and caffeine. Potential salivary biomarkers for diabetes, ovarian cancer, breast cancer, oral cancer, preterm labor and exposure to the coronovirus in severe acute respiratory syndrome (SARS) have been the focus of several research activities in recent years. With the remarkable advances in areas such as gene therapy (Voutetakis and others, 2004) and gene mapping, saliva will continue to be a source of opportunities for scientific progress used in risk assessment, disease prevention and development of therapeutic modalities. 

**An Alternative to Classic Treatment: The Biofeedback Method**

At present there are a number of relevant studies that suggest the importance of including biofeedback in dental practice. The ability to monitor muscle activity, and, indirectly, the formation of saliva, seems to represent both an important assessment tool, and a way of treatment by using instruction techniques by biofeedback. 

Biofeedback is a therapeutic method that involves the amplification of a human biological signal, usually presented visually or audibly to sense receptors (the eyes and ears), and designed by nature to the detection of stimuli in the external world (exteroceptive stimuli). The biofeedback method allows both patient and therapist to observe subtle changes in internal activity and provides information (feedback) that can be used to modify these physiological events in a desired direction while monitoring them. Regarding the treatment and elimination of pain, biofeedback training is aimed at reducing inappropriate psycho-physiological stimulation (i.e.: muscle inhibition).

Briefly, the biofeedback method can be described as the placement of electrodes over the masseter muscles on each side of the face and connecting these electrodes to the equipment that provides a measure of the summed electrical activity produced by the group of muscles, indicating the clenching of the teeth through an amplifier and a speaker that triggers the alarm.

Surface electromyography (sEMG) is a noninvasive technique that places relatively large recording electrodes on the surface of the skin or over a muscle or muscles of interest. EMG measures muscle activity by detecting electrical potential changes associated with muscle action potential.

Technological advances allowed a miniaturization of some very sensitive monitoring devices capable of real-time monitoring of the muscles of cranio-cervical-mandibular system, transforming this into a straightforward office procedure. The treatment for the temporomandibular disorders (TMD), including the use of sEMG biofeedback training, represents a conservative approach, that is both more convenient than traditional treatments and a reversible procedure.
